# Lower early-stage rectal cancer surgical approaches: therapeutic options and cancer biomarker alterations

**DOI:** 10.3389/fsurg.2025.1656343

**Published:** 2025-09-29

**Authors:** Jian Liu, HongJian Gao, LiHua Wang, Yuan Yao, XingDong Li, Bin Yue

**Affiliations:** Department of Colorectal Surgery, Shenyang Coloproctology Hospital, Shenyang, Liaoning, China

**Keywords:** early-stage rectal cancer, simplified transanal excision (sTE), ruiyun procedure for hemorrhoids (RPH), transanal endoscopic microsurgery (TEM), postoperative complications, tumor recurrence markers

## Abstract

**Background:**

Low early-stage rectal cancer (LeREC), typically referring to pTis and pT1 tumors without nodal involvement or lymphovascular/perineural invasion and confined to the mucosa, can potentially be treated with transanal local excision techniques. While transanal endoscopic microsurgery (TEM) remains a common minimally invasive option, it is technically demanding and costly. There remains a need for safer, simpler, and more accessible alternatives.

**Objective:**

To evaluate the safety and efficacy of a simplified transanal excision (sTE) combined with the Ruiyun procedure for hemorrhoids (RPH) compared to conventional TEM in the treatment of LeREC.

**Methods:**

In this randomized, controlled study, 48 patients with LeREC located within 12 cm of the anal verge were assigned to receive either TEM (*n* = 20) or sTE combined with RPH (*n* = 28). Surgical outcomes and postoperative complications were compared. Immunohistochemical analyses of CDK2, CDK4, CDK6, FOXD1, and PAK4 were performed on primary tumor tissues to investigate potential biomarkers associated with tumor recurrence.

**Results:**

All patients were followed up for 12 months. The sTE + RPH group showed reduced intraoperative bleeding, lower surgical costs, and fewer complications compared to the TEM group. Expression levels of CDK2/4/6, FOXD1, and PAK4 were observed to vary between groups and were potentially associated with recurrence risk.

**Conclusion:**

The combination of sTE and RPH may offer a safe, cost-effective, and feasible alternative to TEM for treating LeREC, particularly in resource-limited settings. It facilitates wider clinical application without compromising curative efficacy.

## 1 Introduction

Colorectal cancer (CRC), encompassing both colon and rectal cancer, remains the fourth leading cause of cancer-related mortality worldwide (1). Rectal cancer (RC) accounts for approximately 30% of newly diagnosed CRC cases, and despite recent advances in its multidisciplinary management-including screening, early diagnosis, surgical resection, radiotherapy, and chemotherapy-clinical outcomes still require substantial improvement (2, 3). Enhancing strategies for early diagnosis and optimizing surgical interventions, particularly in tumors located in the lower two-thirds of the rectum, is essential to reduce locoregional recurrence and distant metastasis, thereby improving overall survival and quality of life for patients (4).

Total mesorectal excision (TME) is considered the gold standard for rectal cancer surgery, offering improved local control and survival benefits. However, it is also associated with considerable morbidity, including sexual, urinary, and bowel dysfunction (5, 6). As a result, TME may not be the most appropriate initial treatment option for patients with early-stage rectal cancer, particularly those with low rectal tumors. Organ-preserving surgical strategies, tailored to tumor stage and location, have therefore gained increasing attention (7, 8). Among these, transanal local excision techniques such as transanal endoscopic microsurgery (TEM), simplified transanal excision (sTE), and endoscopic resection are particularly relevant for early-stage lesions located 5–12 cm from the anal verge.

Early-stage rectal cancer includes pTis lesions, which are confined to the mucosa, and pT1 tumors, which infiltrate the submucosa without lymphovascular invasion. These lesions may be amenable to local excision strategies such as TEM and sTE (9). TEM offers enhanced visualization and precision but requires specialized equipment and expertise. In contrast, sTE is a simplified, cost-effective approach with a lower technical threshold, making it suitable for use in primary care settings. When combined with the Ruiyun procedure for hemorrhoids (RPH), sTE may also reduce postoperative complications and preserve anal sphincter function, particularly in patients with coexisting hemorrhoids (10).

In addition to refining surgical techniques, molecular biomarkers may provide valuable insights into tumor biology, thereby informing treatment decisions and prognosis. Cyclin-dependent kinases (CDKs), including CDK2, CDK4, and CDK6, are central regulators of cell cycle progression and proliferation (11–14). The P21-activated kinase 4 (PAK4), which is a member of the serine/threonine protein kinase family, can promote cancer cell proliferation and migration (15–17). The serine/threonine kinase PAK4 is implicated in tumorigenesis and metastasis through its regulation of cytoskeletal dynamics and cell migration (18). Similarly, FOXD1, a member of the forkhead transcription factor family, is associated with epithelial-to-mesenchymal transition (EMT) and poor prognosis across multiple cancer types (19, 20).

This study aimed to compare the clinical outcomes and molecular characteristics of patients with low early-stage rectal cancer (LeREC) treated with either TEM or sTE combined with RPH. By evaluating the expression of key proliferation and invasion markers, including CDK2, CDK4, CDK6, PAK4, and FOXD1, we sought to provide further evidence to support safe, effective, and accessible surgical management strategies for early-stage rectal cancer.

## 2 Material–methods

### 2.1 Study design

This prospective, randomized, single-blind, parallel-controlled trial was designed to evaluate the efficacy and clinical applicability of two modified local excision techniques for low early-stage rectal cancer (LeREC). Eligible patients were randomly assigned to either the treatment group, which underwent a combination of simplified transanal excision (sTE) with the Ruiyun procedure for hemorrhoids (RPH), or the control group, which received standard transanal endoscopic microsurgery (TEM). All patients were followed for 12 months postoperatively to assess surgical outcomes and recurrence. The trial protocol was conducted in accordance with the CONSORT 2017 guidelines and the SPIRIT 2013 recommendations for interventional trials.

## 3 Surgical procedures

### 3.1 Transanal endoscopic microsurgery (TEM)

**Figure 1:**
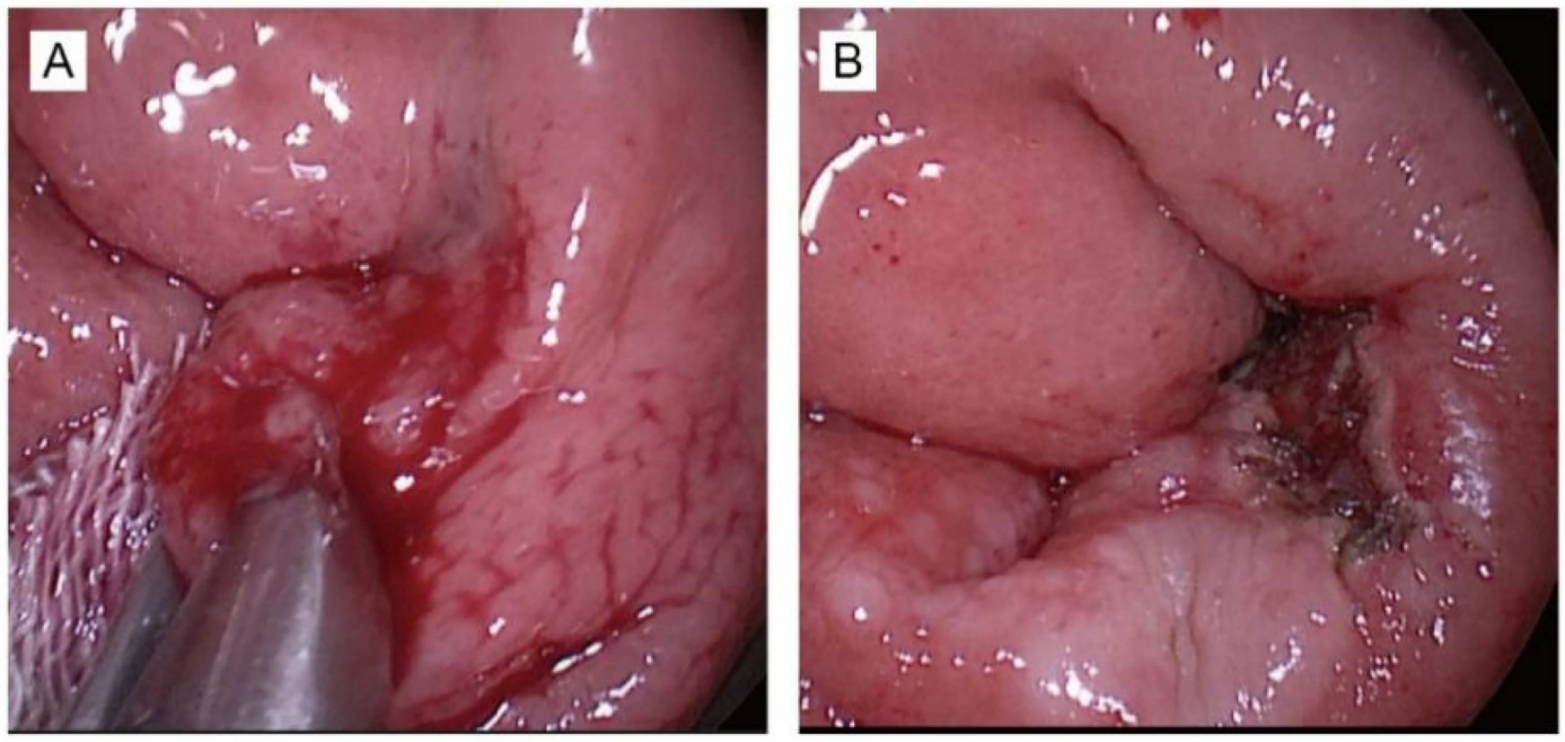
This photograph demonstrates the surgery of TEM. **(A)** The photograph before tumor resection. **(B)** The photograph after tumor resection.

Patients in the control group underwent standard transanal endoscopic microsurgery (TEM) under general anesthesia. A high-frequency electrosurgical knife was employed to delineate the tumor margin, maintaining a distance of approximately 10 mm from the visible edge of the lesion. The lesion was then excised in full under endoscopic visualization using an electric scalpel to ensure complete resection with clear margins. The resected tissue samples were collected and submitted for histopathological and immunohistochemical analysis (Figures 1A,B).

### 3.2 Simplified transanal excision (sTE) combined with Ruiyun procedure for hemorrhoids (RPH)

**Figure 2:**
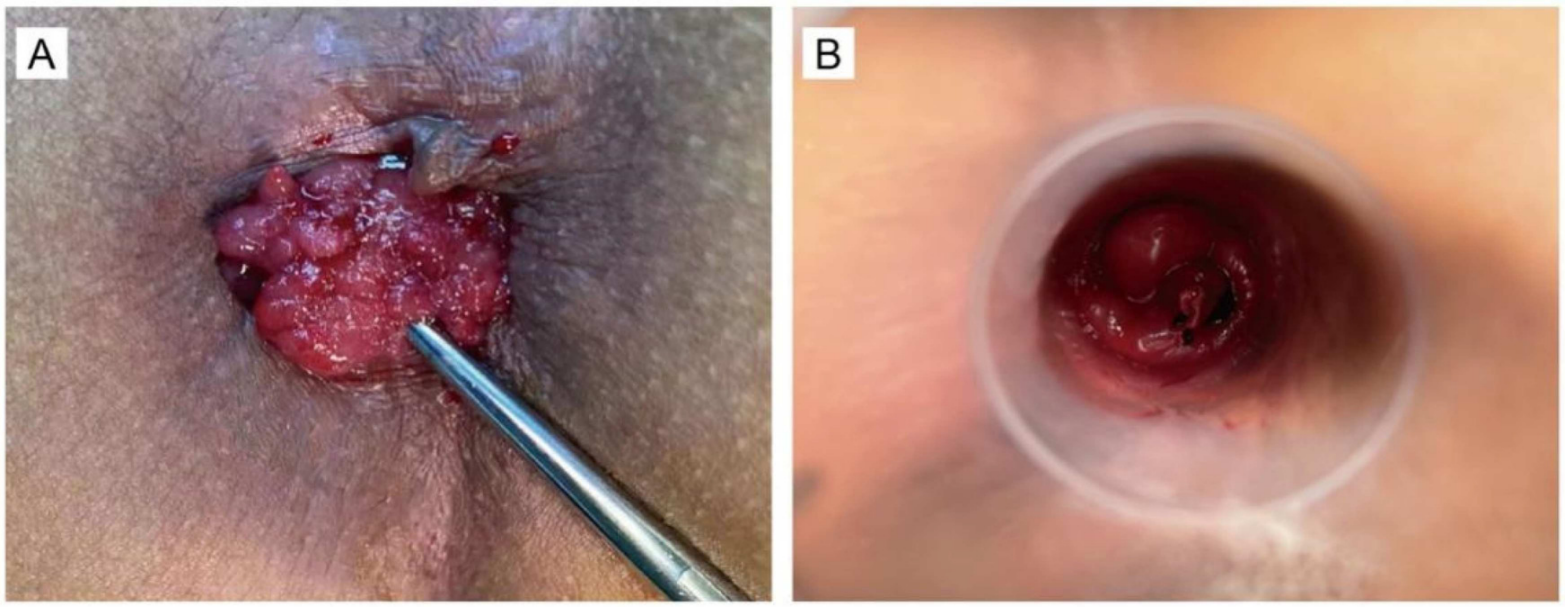
This photograph demonstrates the surgery of combination sTE with RPH. **(A)** The photograph before tumor resection. **(B)** The photograph after tumor resection.

Patients in the treatment group received a combined procedure involving simplified transanal excision (sTE) and the Ruiyun procedure for hemorrhoids (RPH), designed to facilitate local tumor removal while preserving sphincter integrity. The RPH system was applied to deliver controlled negative pressure (0.08–0.1 MPa) to the tumor site, followed by elastic ligation. Once the lesion was securely ligated, it was excised. This method was developed to simplify the surgical workflow and minimize intraoperative bleeding and postoperative complications. All excised specimens were subsequently evaluated using routine histopathological and immunohistochemical techniques (Figures 2A,B).

## 4 Ethics

This study was approved by the Clinical Research Ethics Committee of Shenyang Coloproctology Hospital. All procedures involving human participants were conducted in accordance with the ethical standards of the institutional and/or national research committee, and with the 1964 Declaration of Helsinki and its later amendments. Written informed consent was obtained from all participants or their legal guardians prior to study enrollment.

## 5 Patient eligibility

### 5.1 Diagnostic criteria

The diagnosis of early-stage rectal cancer was established according to the Chinese Guidelines for the Diagnosis and Treatment of Colorectal Cancer (2022). All patients were histologically confirmed by pathological examination of resected tumor tissues and peri-tumoral tissues (minimum distance of 3 cm from the tumor margin).

### 5.2 Inclusion criteria

Eligible patients were required to meet all of the following criteria:

1. Histologically confirmed early rectal cancer (pTis or pT1) with a maximum tumor diameter ≤ 3.0 cm
2. Tumors with high or moderate differentiation
3. Tumor invasion confined to the mucosal or submucosal layers
4. No evidence of lymphovascular or perineural invasion, and no distant metastases
5. Tumor located ≤ 12 cm from the anal verge
6. Ability to provide informed consent and willingness to comply with study protocol

### 5.3 Exclusion criteria

Patients were excluded if they met any of the following conditions:

1. Tumor diameter > 3.0 cm or histological evidence of poorly differentiated carcinoma
2. Presence of anal or rectal stenosis
3. Tumor infiltration involving the muscularis propria, or evidence of lymph node or distant metastasis
4. Co-existing irritable bowel syndrome with severe constipation or diarrhea
5. Any medical condition contraindicating surgical intervention
6. Pregnancy or lactation

### 5.4 Simple randomization and blinding

Eligible patients were randomly assigned in a 1:1 ratio to either the treatment group (sTE combined with RPH) or the control group (TEM) using a simple randomization method. Randomization was performed using the RANDBETWEEN function in Microsoft Excel to generate 48 random integers (either 1 or 2), each corresponding to one of the two treatment arms, thereby ensuring equal probability of assignment to either group. The random numbers were sealed in opaque envelopes, and each patient who met the inclusion criteria selected one envelope to determine group assignment. Blinding was maintained as follows: only the surgeons performing the procedures were aware of the group allocation. Patients, anesthesiologists, data collectors, data analysts, and other study personnel remained blinded to the treatment assignments throughout the trial to minimize potential bias. The study protocol was designed and supervised by experienced clinical researchers with rigorous quality control to ensure methodological integrity.

### 5.5 Tissue collection

A total of 48 rectal cancer tissue specimens were collected from patients with early-stage rectal cancer localized in the lower two-thirds of the rectum who underwent surgical resection at Shenyang Coloproctology Hospital. Among them, 20 patients received transanal endoscopic microsurgery (TEM), and 28 underwent a combination of simplified transanal excision (sTE) with the Ruiyun procedure for hemorrhoids (RPH). All tissue samples were pathologically confirmed as early-stage rectal cancer (pTis or pT1), well-differentiated, with no evidence of lymphovascular or perineural invasion or distant metastasis. Peri-tumoral tissues were also sampled at least 3 cm from the tumor margin.

### 5.6 Immunohistochemistry

Tissue samples were fixed in 4% formalin, embedded in paraffin, and sectioned at a thickness of 5 μm. The study protocol was approved by the Ethics Committee of Shenyang Coloproctology Hospital and conducted in accordance with the Declaration of Helsinki.

Tissue sections were deparaffinized, rehydrated through graded ethanol (100%–75%), and subjected to immunohistochemical staining using a streptavidin-peroxidase (S-P) kit (Maixin Biotechnology Co., Ltd., Fuzhou, China). Primary antibodies included: anti-CDK2 (#18048, 1:200, Cell Signaling Technology), anti-CDK4 (#12790, 1:200, CST), anti-CDK6 (#13331, 1:100, CST), anti-PAK4 (#52694, 1:400, CST), and anti-FOXD1 (ab129324, 1:50, Abcam). Sections were incubated with primary antibodies overnight at 4 °C, followed by secondary antibody incubation for 1 h at room temperature. Detection was performed using streptavidin-peroxidase complex and diaminobenzidine (DAB; Maixin Biotechnology). Slides were counterstained with hematoxylin, dehydrated, and mounted for microscopic examination.

### 5.7 Statistical analysis

All statistical analyses were performed using SPSS version 17.0 software (SPSS Inc., Chicago, IL, USA). Categorical variables were analyzed using the Chi-square (*χ*²) test and presented as frequencies and percentages. Continuous variables were expressed as mean ± standard deviation (SD). For normally distributed data, comparisons between two groups were conducted using the independent samples Student's *t*-test. For non-normally distributed data, the Mann–Whitney *U*-test was applied. A *p*-value of <0.05 was considered statistically significant.

## 6 Results

### 6.1 Enrollment and randomization of LeREC patients

**Figure 3:**
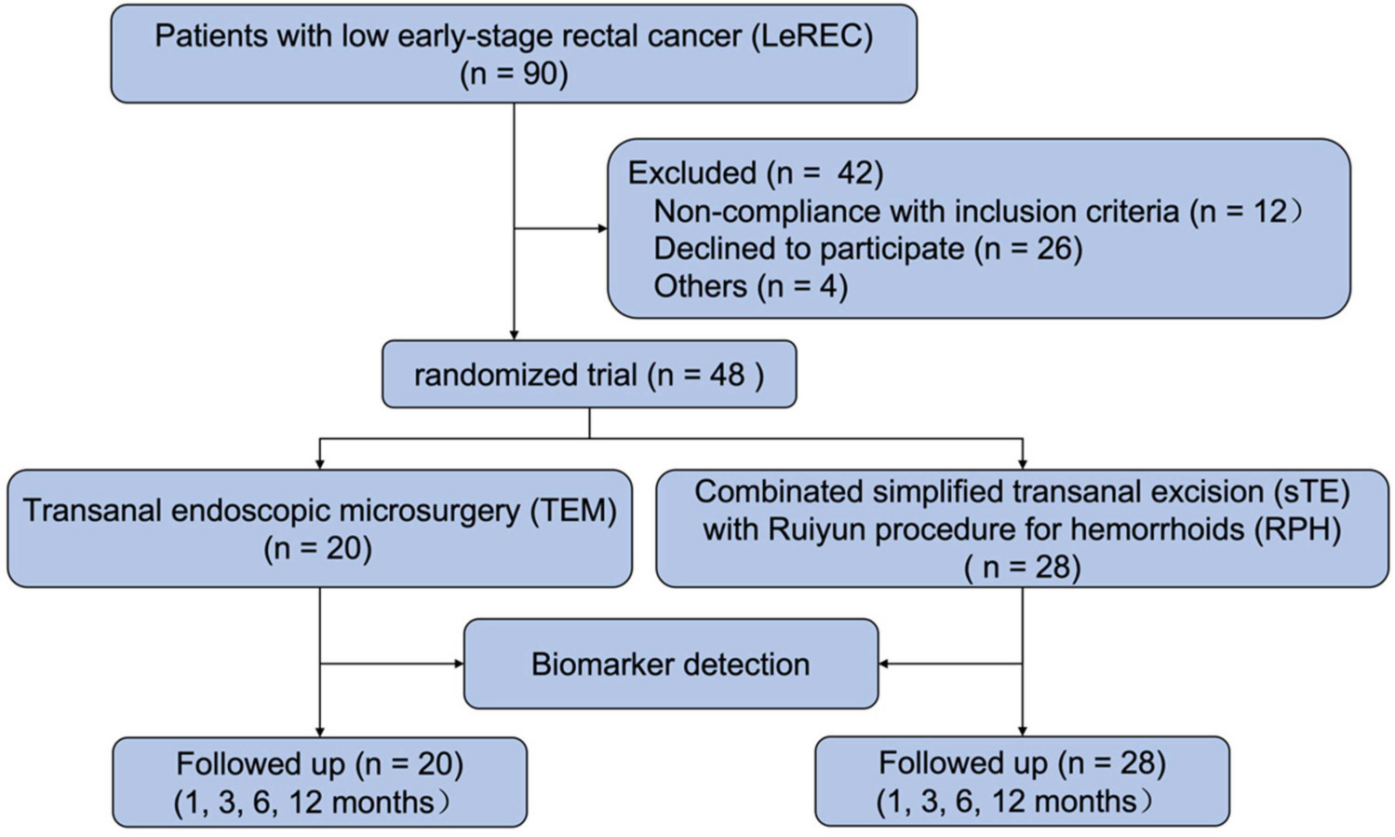
Flowchart of the inclusion, randomization and management of the study subjects in the LeREC.

Between February 1, 2015, and December 31, 2019, a total of 90 eligible patients diagnosed with low early-stage rectal cancer (LeREC) were assessed for enrollment at Shenyang Coloproctology Hospital (Shenyang, China). Of these, 48 patients met the inclusion criteria and provided informed consent for participation in the randomized controlled trial, while 42 patients were excluded based on predefined exclusion criteria. A total of 20 patients were randomized to undergo transanal endoscopic microsurgery (TEM), and 28 patients were assigned to receive simplified transanal excision (sTE) combined with Ruiyun procedure for hemorrhoids (RPH) (Figure 3).

### 6.2 Patient characteristics and surgical outcomes

The final follow-up date was October 20, 2021. None of the patients had received chemotherapy or radiotherapy prior to surgical intervention. The baseline clinical characteristics of all 48 patients diagnosed with low early-stage rectal cancer (LeREC) are summarized in Table 1. Among them, 25 were male and 23 were female, with a median age at surgery of 56 years (range: 32–77 years). This study was reviewed and approved by the Ethics Committee of Shenyang Coloproctology Hospital. Written informed consent was obtained from each participant or their legally authorized representative prior to enrollment.

**Table 1:** The patient characteristics of lower two-thirds of early-stage rectal cancer patients who underwent TEM surgery or sTE & RPH surgery.

Characteristic	TEM (20)	sTE & RPH (28)	*P*
Age			
≥60	14 (70%)	17 (60.7%)	0.507
<60	6 (30%)	11 (39.3%)	
Gender			
Male	12 (60%)	13 (46.4%)	0.353
Female	8 (40%)	15 (53.6%)	
Tumour diameter (≤30 mm)			
≥15	4 (20%)	9 (32.1%)	0.351
<15	16 (80%)	19 (67.9%)	
TNM pT stage			
pTis	15 (75%)	20 (71.4%)	0.784
pT1	5 (25%)	8 (28.6%)	
Distance from the anal verge (≤5 cm)			
≥3	9 (45%)	16 (57.1%)	0.406
<3	11 (55%)	12 (42.9%)	

Twenty patients underwent transanal endoscopic microsurgery (TEM). The mean operative time was 80 min (range: 60–90 min), with an average intraoperative blood loss of 46 ml (range: 30–90 ml). The average postoperative hospital stay was 7 days (range: 5–8 days). In contrast, 28 patients received the combined simplified transanal excision (sTE) with Ruiyun procedure for hemorrhoids (RPH). The mean operative time was 30 min (range: 25–45 min), with average blood loss of 15 ml (range: 10–30 ml). The average postoperative hospital stay in this group was 5 days (range: 3–7 days). As TEM requires high-flow carbon dioxide (CO_2_) insufflation (8–16 mmHg) to dilate the rectal lumen, regular bowel function recovery took significantly longer (median 70 days, range: 45–90 days) compared to the sTE + RPH group (median 20 days, range: 8–40 days) (Table 2).

**Table 2:** Intraoperative and postoperative characteristics of lower two-thirds of early-stage rectal cancer patients who underwent TEM surgery or sTE & RPH surgery.

Characteristic	TEM (20)	sTE & RPH (28)
**Intraoperative**		
Estimated blood loss (ml)	46 (30–90)	15 (10–30)
Operating time (min)	80 (60–90)	30 (25–45)
Sewn (Stitch count)	10–15	5–10
Postoperative		
Pain (Vax)	1–5	0–2
Postoperative length of stay (day)	7 (5–8)	5 (3–7)
Early morbidity		
Wound infection (*n*)	3	0
Pelvic abscess (*n*)	2	1
Perforation (*n*)	1	0
Late morbidity		
Anastomotic strictures (*n*)	5	1
Bleeding after scabs (*n*)	3	1
Bowel training time (day)	70 (45–90)	20 (8–40)
Anal fissure (*n*)	2	0

### 6.3 Biomarker expression in tumor tissues

**Figure 4:**
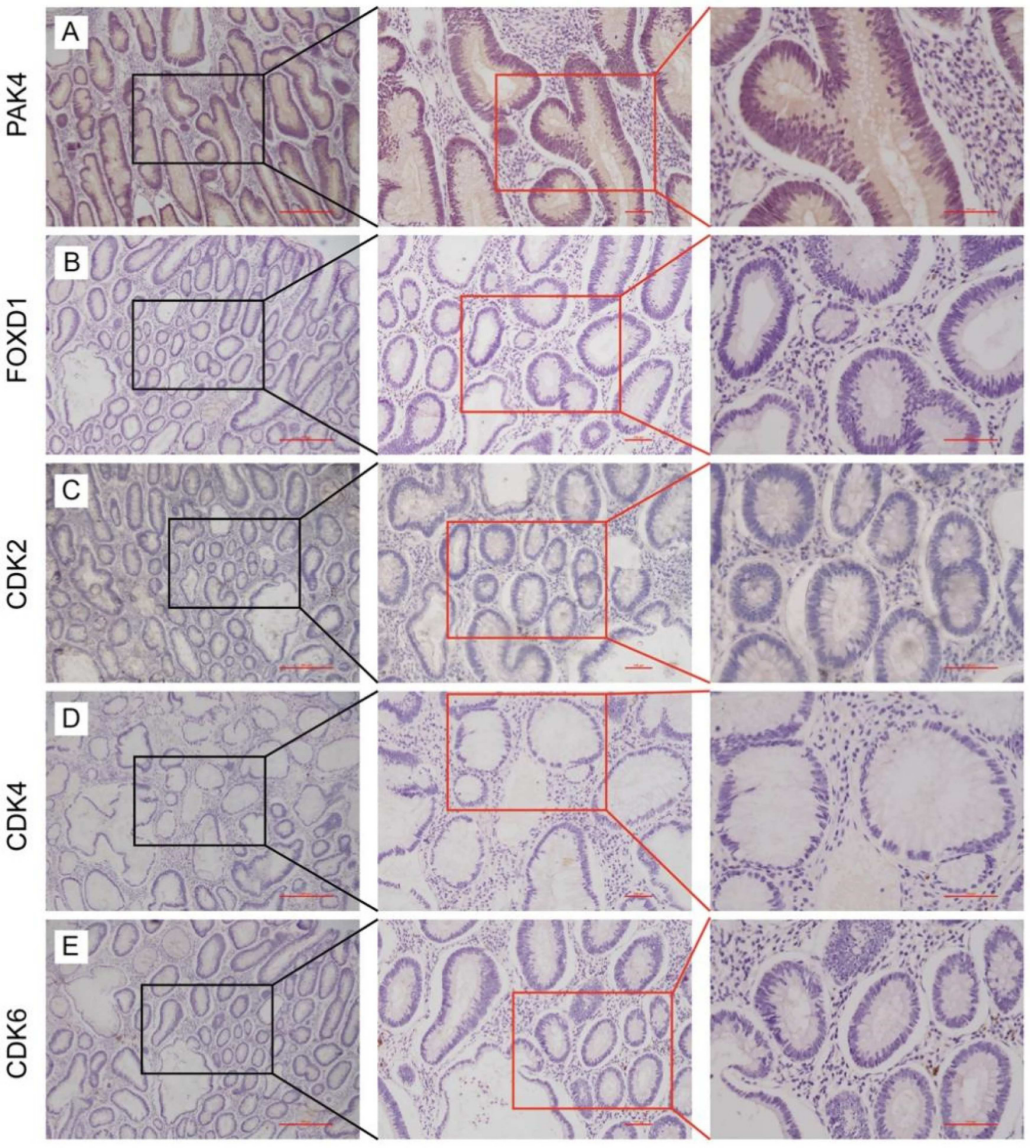
Expression of PAK4 **(A)**, FOXD1 **(B)**, CDK2 **(C)**, CDK4 **(D)**, and CDK6 **(E)** in low early-stage rectal cancers by using immunohistochemistry analysis.

To assess the invasive and metastatic potential of low early-stage rectal cancer, we evaluated protein expression levels of PAK4 and FOXD1 via immunohistochemical staining (Figures 4A,B). Furthermore, to confirm the proliferative capability of tumor cells, expression levels of cyclin-dependent kinases CDK2, CDK4, and CDK6 were analyzed (Figures 4C–E). Representative serial section images were obtained at various magnifications. Notably, all analyzed biomarkers showed negative expression in tumor tissues, indicating low proliferative and metastatic activity in these early-stage tumors.

## 7 Discussion

For patients with malignant rectal lesions, organ-preserving surgical strategies are increasingly favored in the management of low early-stage rectal cancer (LeREC). Selection of an appropriate surgical approach is essential to balance oncologic control with preservation of function, minimization of complications, and reduction of healthcare costs (21). In this prospective, randomized, single-blind, parallel-controlled trial, we compared two surgical approaches—transanal endoscopic microsurgery (TEM) and simplified transanal excision (sTE) combined with the Ruiyun procedure for hemorrhoids (RPH)—across multiple clinical parameters including operative time, intraoperative bleeding, postoperative bowel function recovery, hospitalization duration, procedural cost, and feasibility in non-tertiary hospital settings.

Our findings suggest that sTE combined with RPH offers several potential advantages over TEM, including reduced blood loss, shorter surgical time, quicker return to bowel function, and lower overall cost. Moreover, the technique requires less specialized equipment and training, making it more accessible to lower-level healthcare institutions. In contrast, TEM is highly dependent on surgeon expertise and institutional resources, and while it remains a gold standard for local excision of well-differentiated, early-stage rectal tumors, its broader application may be limited by practical constraints.

Oncological safety remains the primary concern in the surgical treatment of rectal cancer. Complete excision of tumor tissue, prevention of local recurrence, and accurate pathological assessment are crucial for long-term disease control. In this study, we further evaluated tumor biology using immunohistochemical analysis of key molecular markers. PAK4 and FOXD1 have been implicated in promoting tumor invasion and metastasis, whereas CDK2, CDK4, and CDK6 are central to cell cycle regulation and tumor cell proliferation. These findings suggest that molecular profiling could complement histopathologic evaluation in identifying patients at higher risk of recurrence and in selecting candidates for conservative surgical approaches.

Immunohistochemistry has emerged as a practical and informative tool in the evaluation of oncogene expression in rectal cancer tissues. The lack of positive expression for PAK4, FOXD1, and CDK2/4/6 in our cohort suggests low proliferative and invasive potential, supporting the suitability of organ-preserving surgery in carefully selected LeREC patients. Moreover, this multi-marker approach may provide a refined molecular stratification framework for personalized treatment planning in the future.

In conclusion, our study provides evidence supporting the use of sTE combined with RPH as a safe, effective, and resource-efficient surgical option for LeREC. The integration of molecular biomarker analysis into routine clinical practice could enhance patient selection and prognostic accuracy, ultimately guiding surgical decision-making and improving outcomes.
